# Back to living well: community-based management of low back pain: a feasibility study

**DOI:** 10.1186/s40814-021-00863-7

**Published:** 2021-06-24

**Authors:** Luciana G. Macedo, Julie Richardson, Michele C. Battie, Mark Hancock, Matthew Kwan, Genevieve Hladysh, Linda Zhuo

**Affiliations:** 1 School of Rehabilitation Science, Faculty of Health Sciences, McMaster University, Hamilton, Ontario Canada; 2 School of Physical Therapy and Western’s Bone and Joint Institute, Western University, London, Ontario Canada; 3 Macquarie University, Sydney, New South Wales Australia; 4 Department of Family Medicine, Faculty of Health Sciences, McMaster University, Hamilton, Ontario Canada; 5 YMCA Hamilton, Burlington, Brantford, Canada; 6 Western University, London, Ontario Canada

**Keywords:** Low back pain, Recurrence, Flare up, Physical activity, Education, Self-management, Community intervention, Feasibility

## Abstract

**Background:**

Low back pain (LBP) is a long-term health condition with an unpredictable pattern of symptomatic episodes, remission, and recurrence. Recently published systematic reviews suggest that exercise is the most effective intervention for preventing recurrences of LBP in persons that have recovered. Similar programs may also be effective in preventing flare-ups in persistent LBP. The aim of this study was to test the feasibility of the *Back to Living Well* program (Physical activity + Education + Self-management) developed to prevent recurrence or flare-ups of LBP. The study evaluated feasibility in terms of recruitment rate, adherence, satisfaction with the exercise and education sessions, and the data collection procedures. We also aimed to evaluate barriers and facilitators to the engagement in the program.

**Methods:**

Seventeen participants with non-specific LBP recently discharged from care from physiotherapy, chiropractors or physician care (< 3 months) were referred to the study by health care providers or community advertisements between December 2018 and February 2019. Participants underwent a 12-week (1 session/week) individualized, group-based exercise in the community and 4 sessions (30 min each) of education. All participants completed an action plan weekly for 12 weeks and wore an activity monitor for 6 months. All participants responded to weekly pain measures and completed study questionnaires at baseline, 3- and 6-months. Feasibility outcomes included recruitment, attrition rates and satisfaction. At the end of the intervention, participants completed an end-of-program survey.

**Results:**

Twenty-nine participants were screened for eligibility; 20 were deemed eligible, while 17 were included over a 2-month period meeting our feasibility targets. In total, 16 completed follow-up study questionnaires at 3 months, and 15 completed the 6-month follow-up. Fourteen participants responded to weekly messages, while 3 participants reported not having a mobile device or Internet access. In total, 15 participants responded to our end-of-program survey. Average age was 54.9 (11.7); 9 were female (53%), and the mean duration of LBP was 62.9 (69.7) months. All satisfaction responses in relation to the exercise program, education program and data collection procedures reached our threshold of 70% out of 100%. Reported barriers to engagement in the program included fear of injury, lack of motivation and travel. Facilitators included proximity to home, low cost, flexible schedule and friendly location.

**Conclusion:**

The results show the program is feasible in terms of recruitment, low attrition, and patient satisfaction. Participants highlighted the excellent, relevant education program and the positive, personalized exercise. Future studies should evaluate the effectiveness of this intervention within a fully powered randomized controlled trial.

**Trial registration:**

NCT03328689

**Supplementary Information:**

The online version contains supplementary material available at 10.1186/s40814-021-00863-7.

## Key messages on feasibility

- We were uncertain if participants could be recruited and retained through the course of the study. We were also uncertain whether the new intervention was feasible and whether patients would be satisfied with the mode of delivery, the content of the education and the exercise or the program in general. We were also uncertain whether collecting outcomes using REDCap, mobile app and activity monitors would be feasible.
- Recruitment rate was lower than expected; however, within feasibility parameters with potential increase in advertisement outreach. The retained rate for the intervention and the follow-ups were within feasibility parameters. There was high satisfaction with the exercise and education program demonstrating the intervention to be highlight feasible. Finally, the response rate of the app-generated question was feasible with high response rates for those that owned a mobile device and not those that did not own their own device.
- Recommendations are made to increase resources to improve recruitment rate and potentially increase the study timeline when planning for a larger study. Further, provision of mobile devices for completion of weekly app-related questions or the inclusion of participants that own a mobile device may be required.

## Background

Longitudinal studies of the natural history of low back pain (LBP) have defined the contemporary view that back pain is typically a long-term health condition with an unpredictable pattern of symptomatic episodes, remission and recurrence [1–3]. After an acute episode of LBP, approximately 60% of individuals recover within 3 months [4, 5] (pain-free for at least 1 month [4, 5]), but of those, 50–70% experience a recurrence [6] (episode of pain lasting at least 24 h following recovery [1, 6]) within 1 year. For persons who never recover fully, 60% will have at least one pain flare-up within 1 year [1] (worsening of the condition that lasts from hours to weeks that is difficult to tolerate and may impact usual activities and emotions [7]). This means that regardless of whether individuals have recovered or not from an episode of LBP, about 60% will have a recurrence or an activity limiting flare-up within the following year. This fluctuating nature of low back pain is a major reason why the condition carries such a large global social and economic burden [8].

Exercise therapy is the most commonly endorsed treatment for an episode of chronic/persistent LBP in clinical practice guidelines [9, 10] and systematic reviews [11]. Among the wide variety of exercise approaches available, there is no evidence of superiority of one exercise over another [9]. Evidence also supports exercise as effective in preventing recurrences of LBP [12, 13]. A recent systematic review found exercise combined with education (typically delivered in a group) reduces the risk of a recurrent episode of back pain by 45% within a year compared with no treatment or minimal intervention [12, 13]. In addition, exercise reduces the length and intensity of recurrent episodes when they occur [12, 13]. A review by Choi et al. found exercise programs delivered during an episode of back pain were not sufficient to prevent recurrences, but programs targeting exercises after discharge from care were more effective in preventing recurrences [12]. While methodologically sound, these studies with promising results have been primarily focused on persons that have recovered from a previous LBP episode. Since recent evidence suggests that persons with chronic LBP (pain > 3 months [9]) also suffer from activity-limiting and -disabling flare-ups [1, 2, 14], the prevention of flare-ups in persons with chronic or recurrent LBP is as important as preventing recurrences in persons who are pain free. To our knowledge, there are no studies that have evaluated whether similar exercise programs are also effective for preventing flare-ups in persons with persistent LBP.

Engagement in physical activity together with associated behavioural changes directed toward a healthier lifestyle are important issues that need to be addressed in the general population. Not surprisingly, barriers to exercise can be intensified in individuals with back pain who often have kinesiophobia (fear of movement), low self-efficacy and unhelpful beliefs about their back pain [8, 15–17]. A fear of recurrence of back pain and flare-ups is one of the main reasons why individuals with back pain avoid physical activity [18, 19]. An education program which addresses outcome expectations, self-management and exercise strategies, such as pacing, is crucial for the success of a physical activity program in this patient group. Thus, the *Back to Living Well* program addresses an important gap between the prescription of exercise by a health care provider and engagement and adherence to physical activity in the community by individuals suffering with back pain. This sustainable community-based physical activity and education program is focused on self-management with features to boost adherence in physical activity [18, 20]. The goal of this program is to support individuals who have recently received care for their LBP and to enable them to act on the health care recommendations and engage in physical activity in the community. The program will address known barriers to continued physical activity.

Given the complexities of designing a community-based program, as well as developing an evaluation program that includes the use of technology for real time data capture, we aimed to conduct a study to test the feasibility of a program of care for patients with LBP. The primary objectives of this study were to test the feasibility of the *Back to Living Well* program for patients with non-specific LBP in terms of recruitment, barriers and facilitators for the engagement in the program, adherence to the program, satisfaction with the exercise and education sessions and data collection procedures.

## Methods

### Study design

This was a one-arm intervention study with 6 months follow-up. The study was registered a priori (NCT03328689) and received ethics approval from the Hamilton Integrated Health Research Board (HiREB #2721).

### Participants and setting

This study was conducted in Hamilton, Ontario, Canada. Patients were recruited from local physiotherapists, chiropractors and osteopaths delivering care in the community or through local YMCA advertisement (e.g. newsletter and social media). Physiotherapists and chiropractors were informed about the study using fax, telephone contact or in-person visits. Identified participants were referred to the study using a standard referral form that included the participant’s contact information, the presence of comorbidities and general exercise recommendations (e.g. contra-indications). Persons identified through advertisement were asked to request a referral directly from their treating health care professional, which could include physiotherapists, chiropractors, osteopaths and physicians. Given our budget constraints, we aimed to recruit 20 participants within 2 months of recruitment. Sample size calculation was not conducted, and a sample size of 20 participants was deemed sufficient as per our previous experience.

*Participants were eligible if they met the following criteria:*

- Have been discharged < 3 months ago from care (e.g. physiotherapy, chiropractic or osteopathic) following a course of treatment for LBP (pain in the area between the 12th rib and buttock crease) [9]
- Have non-specific LBP or a history of non-specific low back pain, meaning the pain is not attributed to a specific diagnosis such as ankylosing spondylitis, vertebral fracture, etc. Non-specific LBP is sometimes called mechanical LBP and accounts for ~ 85% of LBP diagnosis [21, 22]
- Between 18 and 80 years of age
- We included 2 groups of patients: (1) Currently pain-free (had recovered from an episode of LBP; or with mild/moderate LBP (pain intensity ≤ 5/10). The cutoff of 5/10 is used in the literature to dichotomise low/medium-to-high pain intensity [2, 23]
  *Participants were excluded if they met the following criteria:*
- Comorbidity preventing participation in physical activity. The Physical Activity Readiness Questionnaire (PAR-Q) from the American College of Sports Medicine guidelines [24] was used for screening and further confirmed through contact with the referring health care professional.
- Inadequate English to complete outcome measures
- Currently participating in an exercise program similar to the one being evaluated
- History of spine surgery

### Procedures

Eligibility criteria were initially confirmed by the referring health care professional and later by the research staff conducting baseline assessment. All participants attended an initial appointment during which the research assistant gave an overview of the study, and participants signed consent forms. Baseline questionnaires were completed on the REDCap survey platform through a link sent to the participant’s email address. All outcome measures in the study, apart from the activity monitor, were self-reported. Longitudinal data collection procedures were explained to the participant, and their smartphones were set up to receive study notifications for weekly pain data collection (MetricWire Inc. app). Online study assessments of self-reported questionnaires occurred at baseline, immediately after the intervention at 3 months, as well as a 6-month follow-up. In addition, participants also responded to weekly questions about their pain, activity limitation and their mood using a mobile app (MetricWire Inc.). Participants were also asked to complete an action plan using REDCap survey and the short form International Physical Activity Questionnaire (IPAQ) weekly for the intervention phase of the study (first 12 weeks). Finally, all participants received an activity monitor (Garmin Vivo 3) and were instructed by the research staff on how to sync their monitor with an electronic device (phone, tablet or computer) for the collection of physical activity outcomes on a biweekly basis. Participants who did not comply with the data collection system were contacted and reminded of the procedures and time requirements.

### Intervention

The *Back to Living Well* program was a collaboration with LiveWell, which is a partnership between YMCA Hamilton, Burlington and Brantford, (YMCA HBB), the local health care funder, Hamilton Health Sciences (HHS) and McMaster University. LiveWell programs are focused on health outcomes for persons with chronic conditions, including frailty/illness prevention, self-management, and easing the transition from hospital to community. Our patient-oriented program was developed within the auspice of the LiveWell programs.

Participants met with a LiveWell specialist (kinesiologist), for approximately 45 min following study baseline assessment (conducted by a research personnel). The LiveWell specialist assessed baseline capacity in completing individual exercise goals and designed an individualized exercise program taking in consideration referral recommendations. The referral included recommendations, contraindications and comorbidities relevant to exercise prescription (e.g. knee osteoarthritis).

The 12-week program consisted of a weekly 1-h physical activity session. There were also four 30-min education classes (2 h in total) held in conjunction with the first 4 exercise sessions. While group classes were used, a large portion of the exercises were tailored to each individual. Qualitative studies of patient’s beliefs regarding LBP care support the development of an individualized program within a shared decision-making framework [25–27]. Although classes were once a week, as participants progressed through the program and became more familiar with exercising, they were encouraged to engage in physical activity at least 3× per week (as per WHO recommendations for exercise) within or outside the YMCA. All participants received a 4-month free membership at the YMCA and had access to all exercise facilities and programs.

Evidence from exercise therapy studies [11] and prevention of recurrent episodes in LBP [12, 13] do not provide recommendations on the type of exercises (e.g. strength training). This is in line with clinical practice guidelines that suggest that not one form of exercise is superior to another and that engaging in exercise is what is important [9]. Thus, classes included a 15-min group warm up, (e.g. plyometrics, light walking with arm and leg movements), 30 min of individualized exercises in the gym, which included all 3 types of exercise (cardiovascular, strengthening and stretching) [28] and a 15-min group program consisting of back-specific exercises (e.g. core strengthening) [29, 30], and cool down (consisting primarily of relaxation and mindfulness) [31]. Mindfulness has been cited as an important component for the management of central sensitization and persistent pain [31]. The individualized portion of the exercises targeted at each individual’s goals. For example, if a participant wanted to lose weight or develop muscle mass, the program targeted these goals (e.g. cardiovascular program and strengthening).

Exercises were delivered using a graded activity principle [32]. That is, at the first appointment, the LiveWell specialists identified individual goals and needs based on an interview and referral forms. Baseline capacity and pain levels during the activity were tested. *Initial levels of activity were set at 60–75% of the initial capacity or when pain increased greater than 2 points on a 0–10-point scale (where 0 is no pain and 10 is the worse pain possible).* Then, throughout the program, the activity was gradually progressed to move closer to the desired goal. Progression was set at an increase of approximately 10% per week in either duration, repetitions or load. If a participant experiences a flare-up during the program, it was important to address the problem without an injury focus and potentially modify the program (e.g. alter the intensity).

Education was integrated and comprised evidence-based information on back pain [33, 34], efficient use of the back during daily activities, principles of self-management [35, 36], self-efficacy and pain neurophysiology [37]. The education provided an understanding of back pain and aimed to reduce fears associated with the condition [38]. It also provided participants with self-management strategies, such as pacing, ergonomics and action planning. All participants were encouraged to complete an action plan (activity related) weekly with the support of the LiveWell specialist as part of the self-management component of the intervention. A detailed exercise protocol can be provided upon request.

Strategies to improve long-term adherence included social support (e.g. group exercises in a friendly environment) and the provision of an activity monitor for individual tracking. Social support has been reported in the literature as one of the main tools to boost adherence to exercise programs [17]. Participants were encouraged to use the activity monitor provided to all participants for tracking physical activity as a means of biofeedback and reinforcement. Additionally, activity monitors that use accelerometer technology and track levels of physical activity, steps taken and heart rate have been found to support long-term adherence to physical activity [39, 40].

### Outcomes

#### Primary feasibility outcomes

Feasibility outcomes were recruitment rate, satisfaction with the program, satisfaction with study procedures, adherence with the program and follow-up rates. These data were collected through administrative data and an end-of-study survey. Based on our previous experience, a recruitment rate of 10 participants per month and a retention of 85% were deemed feasible. The survey included questions on satisfaction and enjoyment around the exercise and education programs, how much was learned from the education program, whether they would recommend the program to a friend, and whether the data collection procedures were perceived as bothersome. Survey questions were answered using a visual analogue scale from 0 to 100. Scores of 70 or more were deemed to be important. The survey also included open questions about barriers and facilitators to physical activity and the program that were summarized into a list.

##### Physical activity

We used two methods to evaluate physical activity compliance. These included compliance with the exercise program using the YMCA swipe cards that patients used to enter the facility and weekly physical activity and sedentary behaviour using the activity monitor, Garmin Vivo 3. Data extracted included total steps per week. There is limited information on the psychometric properties of the Garmin activity tracker in a clinical population; however, studies in a general population have demonstrated that the tracker has moderate-to-good reliability, validity and accuracy [41–43].

#### Effectiveness outcomes

##### Disability

The Roland Morris Disability Questionnaire was used to evaluate disability [44]. This questionnaire is one of the most widely used and recommended disability measures in LBP and has been shown to be valid, reliable and responsive with minimal clinical important difference (MCID) of 6 points [44, 45].

##### Pain

Average pain intensity over the last week was measured using a numeric rating scale (NRS) from 0 to 10, where 0 is no pain and 10 is the worse pain possible [44]. This measure is one of the most commonly used measures to assess pain in LBP studies [44] and is also a core outcome measure in LBP with MCID of 2 points [44, 46].

##### Function

Function was assessed using the Pain-Specific Functional Scale (PSFS) [47]. The PSFS requires patients to identify 3 problematic activities and rate these activities on a scale from 0 to 10. The total score is from 0 to 10, as an average of the 3 activities. The PSFS has evidence for validity, reliability and responsiveness in LBP populations with MCID of 4 [48, 49].

##### Health-related quality of life

Health-related quality of life was assessed using the EQ-5D-5L [46, 50] with plans for a future cost-effectiveness analysis study [51]. Descriptive of each question: mobility, self-care, usual activities, pain/discomfort, anxiety/depression will be presented as per questionnaire guidelines.

##### Number of flare-ups

This outcome measure was assessed weekly using a mobile app. App notifications were sent weekly (every Monday morning) for 6 months. A consensus-based flare-up definition was used: a worsening of the condition that lasts from hours to weeks that is difficult to tolerate and may impact usual activities and emotions [7]. Thus, a participant was deemed to have had a flare-up if (a) there was a worsening of pain levels of at least 2 points on a 0–10 scale as per the established MCID of 2 [44] and (b) if the participants fit the criteria for activity-limiting flare-up as measured using an adaptation of item PI9 of the PROMIS item bank (How much did LBP interfere with your day-to-day activities?). A score of 3 or greater (representing somewhat impactful) on a 1–5 scale was deemed to be activity limiting. We evaluated the number of flare-ups, the length of flare-ups and the intensity of flare-ups.

We also used additional questionnaires to characterize the sample in terms of fears, beliefs and attitudes towards their back pain. These included the TAMPA Scale of Kinesiophobia [52], the Pain Self-Efficacy Questionnaire [53] and the Coping Strategies Questionnaire [54].

### Statistical analysis

Descriptive statistics were used to summarize feasibility outcomes. Given that we had 26 weeks of physical activity data using the activity monitor, we conducted a linear mixed model with physical activity as the dependent variable, time as an independent variable and participants as a random factor. Results for effectiveness outcomes were presented to demonstrate trends, but no statistical analysis was conducted. All statistical analyses were conducted using Stata Inc 14.0.

## Results

### Recruitment rate

Recruitment occurred from December 2018 to February 2019 (2 months in total). We received 19 referrals for the study from health care professionals. We also received 10 direct contacts (email or phone call) from participants responding to the advertisement in the community. Of those, 2 were formally referred to the study following a visit to a health care professional. One participant was already exercising 4 times a week at the YMCA, who was therefore excluded. The primary reason for non-eligibility of individuals from the community was not having a recent history of care seeking. Of the 20 participants eligible for inclusion, 3 decided not to enter the program due to time availability. Thus, we included 17 participants in 2 months for a recruitment rate of 9 patients per month, which is slightly lower than the 10 patients per months in our feasibility threshold. Since we were running group classes, we stopped recruitment once we had enough numbers to fill in 2 classes. There was a maximum 4-week staggered start of some participants into the classes. Participants’ demographic characteristics are included in Table 1. Average age was 54.9 years (11.7); nine (53%) were females and the mean duration of LBP was 62.9 (standard deviation 69.7) months.

**Table 1:** Participant characteristics and questionnaire scores at baseline (*n* = 17)

	Score
Characteristic (mean, SD)	
Age (years)	54.9 (11.7)
Weight (kg)	82.9 (18.5)
Height (cm)	1751. (10.2)
BMI	26.9 (4.8)
Duration of low back pain (months)	62.9 (69.7)
Sex (Female) (n (%))	9 (52.94)
Marital Status (n (%))	
Married	14 (82.4)
Divorced	1 (5.9)
Common law	1 (5.9)
Single	1 (5.9)
Occupation (n (%))	
Not working*	4 (23.5)
Working	13 (76.5)
Employment (n (%))	
Full-time full duties	8 (47.1)
Full-time selective duties	1 (5.9)
Part-time full duties	2 (11.8)
Part-time selective duties	2 (11.8)
Not seeking employment	4 (23.5)
Smoking/medication (n (%))	
Smoking	1 (5.9)
Taking painkillers	2 (11.8)
Education (n (%))	
High school diploma	2 (11.8)
Diploma	5 (29.4)
Bachelor’s degree	1 (5.9)
Post graduate degree	3 (17.7)
Other (e.g. professional training)	6 (35.3)
Scale (mean, SD)	
TAMPA scale of Kinesiophobia	33.7 (6.6)
Pain Self-Efficacy Questionnaire	43.9 (13.4)
Coping Strategies Questionnaire	5.6 (8.3)

### Attrition rate

**Fig. 1:**
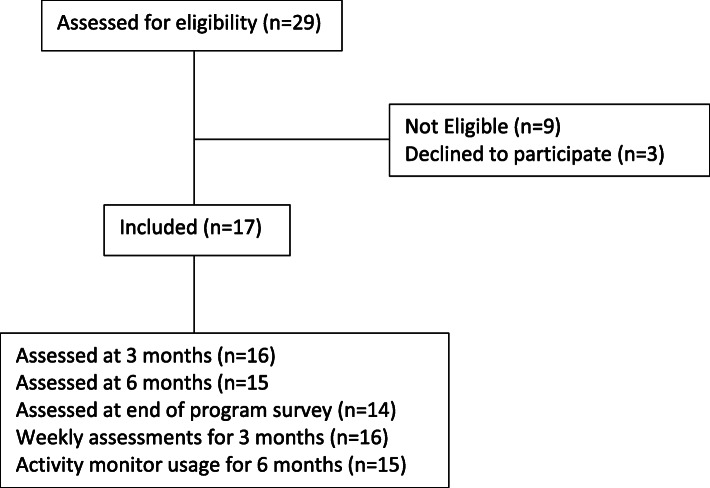
Flow diagram

Of the 17 participants included in the study, all completed baseline assessment, 16 (94%) completed the follow-up assessment at 3 months and 15 (88%) completed the 6-month follow-up meeting, our 85% target for attrition rate. One participant was lost to follow-up at 3 months. Two other participants missed follow-ups, one at 3 months and the other at 6 months. Weekly text messages were responded consistently (at least 80% of all 26 weeks) by 13 (70%) participants. Of the 4 participants that did not respond consistently to weekly messages, 3 did not have regular access to a mobile device or internet to receive notifications. All of these participants, at the beginning of the study, mentioned having access to partners or family devices for the completion of assessments. Interestingly, from all 17 participants, only 1 person did not consistently respond to our weekly REDCap surveys sent to email during the first 12 weeks of the program to complete action planning. Additionally, 15 participants (88%) wore their activity monitors through the 26 weeks of the study with an adherence rate of 70% (wearing and syncing the activity monitor). Finally, 14 participants (82%) responded to the end of program survey. See Fig. 1 for the flow diagram.

### Self-reported outcomes

Participants included in this study had lower-than-average fear of movement and had better self-efficacy and coping strategies than expected, compared with other previously published studies [55]. As expected, participants had lower levels of pain and disability compared to studies including participants seeking care for an episode of LBP (Table 2) [29, 56].

**Table 2:** Baseline, 3rd-month and 6th-month results of outcome measures

	Baseline	3 months	6 months
Scale (mean, SD)			
Weekly Pain Rating (NRS)	3.7 (2.3)	2.7 (1.9)	2.1 (1.8)
Patient-Specific Functional Scale	5.4 (2.8)	6.7 (2.5)	6.9 (6.7)
Roland Morris Disability Questionnaire	6.2 (4.5)	4.8 (3.8)	5.1 (5.7)
EQ-5D-EL			
Mobility	Level 1 = 8 (47%)	Level 1 = 9 (56%)	Level 1 = 9 (64%)
	Level 2 = 6 (35%)	Level 2 = 6 (38%)	Level 2 = 3 (21%)
	Level 3 = 3 (18%)	Level 3 = 1 (6%)	Level 3 = 2 (14%)
	Level 4 = 0	Level 4 = 0	Level 4 = 0
	Level 5 = 0	Level 5 = 0	Level 5 = 0
Self-care	Level 1 = 11 (65%) Level 2 = 4 (24%) Level 3 = 0 Level 4 = 0 Level 5 = 2 (11%)	Level 1 =11 (69%) Level 2 = 5 (31%) Level 3 = 0 Level 4 = 0 Level 5 = 0	Level 1 = 16 (100%) Level 2 = 0 Level 3 = 0 Level 4 = 0 Level 5 = 0
Usual activities	Level 1 = 7 (41%)	Level 1 = 7 (441%)	Level 1 = 8 (57%)
	Level 2 = 7 (41%)	Level 2 = 6 (38%)	Level 2 = 4 (29%)
	Level 3 = 2 (12%)	Level 3 = 2 (12%)	Level 3 = 2 (14%)
	Level 4 = 1 (6)	Level 4 = 1 (6)	Level 4 = 0
	Level 5 = 0	Level 5 = 0	Level 5 = 0
Pain/discomfort	Level 1 = 2 (12%)	Level 1 = 4 (25%)	Level 1 = 5 (36%)
	Level 2 = 6 (35%)	Level 2 = 6 (38%)	Level 2 = 5 (36%)
	Level 3 = 9 (53%)	Level 3 = 6 (38%)	Level 3 = 4 (29%)
	Level 4 = 0	Level 4 = 0	Level 4 = 0
	Level 5 = 0	Level 5 = 0	Level 5 = 0
Anxiety/depression	Level 1 = 10 (59%) Level 2 = 5 (29%) Level 3 = 1 (6%) Level 4 = 1 (6) Level 5 = 0	Level 1 = 9 (56%) Level 2 = 7 (44%) Level 3 = 0 Level 4 = 0 Level 5 = 0	Level 1 = 10 (71%) Level 2 = 4 (29%) Level 3 = 0 Level 4 = 0 Level 5 = 0
Flare-up (number of flare-ups per patient per 3 months)	N/A	0.4 (0.8)	0.35 (0.76)

### Flare

In total, there were 4 participants that had a flare-up during the 23 weeks of the study. One participant had 1 flare-up, 2 participants had 2 flare-ups and 1 participant had 3 flare-ups. Six of the 8 flare-ups occurred during the intervention period of the study. The average amount of change in pain during a flare-up was 2.6 (0.7) points on a 10-point scale. The average change in activity limitation was 1 (0.6) on a 5-point scale. The medium length of flare-ups was for 1 week, with one lasting 3 weeks and one lasting 6 weeks.

### Physical activity adherence

The activity monitors did not show a statistically significant increase in steps over the 6 months of the study when considering participants as random factors; however, there was a significant increase over time when running a simple linear regression (coef = 66.5, 95% CI 9.3 to 123.8, p = 0.023, R^2^ = 0.015). This demonstrates that changes over time are highly depended on each participant. The average number of times a participant attended the YMCA over the 6 months of the study was 13.9 (11.7) ranging from 1 to 40 times, although it is possible that not all participants scanned their cards when entering the facility. Adherence with the education program was tracked, and all participants attended all education sessions or watched education videos online.

### Satisfaction survey

Fifteen of the seventeen participants responded to the survey. All quantitative questions were answered on a visual analogue scale from 0 to 100. All responses reached our threshold for feasibility of 70 points, apart from the question related to bothersome data collection procedures that was 62%. See Table 3.

**Table 3:** Feasibility outcomes

Questions	Responses mean (SD)
How satisfied are you with the program (not at all–extremely)	82.1 (16.5)
How satisfied are you with your exercise outcomes (not at all–extremely)	78.2 (19.6)
How much did you enjoy the exercise program (did not–very much)	78.1 (22.2)
How much did you enjoy the education program (did not–very much)	85.1 (12.4)
How much did you learn from the education program (not at all–a lot)	79.2 (13.3)
Would you recommend this program to a friend or family member? (not at all–definitely)	81.9 (20.4)
How bothersome did you find the data collection process (not at all–extremely)	38.2 (30.3)
How confident are you that you will continue to exercise 150 min per week? (Not at all–extremely)	81.8 (21.1)

In addition to the quantitative questions, participants answered open-ended questions about the program. Participants were asked to report positive aspects of the program, which included many positive comments about the facility, the personnel and the mode of exercise delivery. A summary is presented below:

- Excellent education program
- Enthusiastic trainers that were friendly and helpful
- Good support for the exercise program
- Good to be able to track with activity monitor
- Free membership
- Good communication with YMCA staff
- Opportunity to try out the gym and equipment
- Personal attention and non-threatening atmosphere
- Personalized exercises

Participants were asked to report the negative aspects of the program, which included comments about the data collection procedures and the location of the program. A summary is presented below:

- Do not like that it involves exercise machines.
- Some data collection were confusing and long.
- The location was far, especially in the winter.
- Wearing the activity monitor after 12 weeks of the program
- The time it took to learn about the exercise equipment within a group

Participants were asked to note barriers related to adherence with the exercise program, and many of the highlighted barriers are similar to other previously listed barriers to exercising with LBP [18, 20]. A summary is presented below:

- Fear of injury or pain
- Lack of motivation
- Distance after work in rush hour
- Cost may be a limitation
- Commute and transport

Participants were also asked to note facilitators to adherence with the exercise program. Comments were related to the location, timing and costs. A summary is presented below:

- Low cost
- Close to home
- Flexible schedules
- Friendliness of the location
- Short length of the program and time required

## Discussion

The study results show that the program is feasible in terms of retention rate and patient satisfaction. Our recruitment rate was slightly lower than expected; however, it is still within the parameters for continuing the study with small modifications, specially considering that we reached our study sample within a relatively short recruitment time of approximately 2 months and with few resources to support recruitment. Follow-up rates were higher than 85% for the effectiveness outcomes, with only one participant withdrawing from the study for unknown reasons. Despite the burden of weekly reporting, 70% compliance was reached, which was even higher (85%) among those who owned a mobile device. The use of activity monitors was also found to be feasible to collect physical activity data within the first 12 weeks of the intervention, though longer-term compliance was lower following the cessation of the intervention. Finally, we reached all targets for satisfaction with the program, with the one exception of data collection method (questionnaires, weekly texts/app, and activity monitors) for the exercise program that was perceived as bothersome by a large minority (38%) of participants.

Overall, participants were responsive to the multidimensional components of the LiveWell program. The result of the qualitative open-ended questions demonstrated that participants enjoyed the opportunity to join a community-based exercise program. Many highlighted the importance and the quality of the education program. An important aspect that should be considered in community-based programs is the environment in which these programs are delivered. Many participants highlighted the friendliness of the staff and the non-threatening environment as a significant positive aspect of the program. These factors may also be linked to social support that is one of the main predictors of long-term adherence to exercise and behavioural change [17]. Personalized attention was another factor that was highlighted as being important. This confirms results from qualitative studies of perceived beliefs about LBP care that support the importance of an individualized program [25–27].

Barriers to the engagement in exercise included the long commute and class schedule. Classes were offered in one location only during two specific times (afternoon or early evening). Thus, a larger-scale implementation of this program should consider a greater range of appropriate locations and times for the program. We had an adequate recruitment rate. However, including persons who were recently discharged from care (< 3 months) from physiotherapy and chiropractic clinics limited recruitment to persons with access to these services. Many Canadians do not have access to outpatient rehabilitation as this is not included within the public health system. Thus, broader inclusion criteria could improve on the generalizability of the study capture additional persons living in the community with LBP who would benefit from a community-based program. This includes the potential inclusion of participants with a history of recurrent LBP as well as participants with higher levels of pain that were excluded from this study. These groups represent a large proportion of individuals living with back pain in the community [1, 4, 57].

Tracking YMCA attendance was suboptimal as it is likely that not all participants scanned their cards when entering the YMCA. Thus, tracking attendance of each class for each participant by those delivering the intervention may provide better adherence data. The use of the activity monitors was an efficient method of tracking physical activity levels, although the 70% response rate was a limitation to the information provided. However, there are limitations with this form of physical activity assessment, as some participants engaged in cycling exercises that were not captured by the activity monitor. The same limitation applies to water-based activities. Thus, a higher-grade activity tracker in combination with diaries may provide a better representation of physical activity performance as well as adherence.

Weekly follow-ups were limited by the availability of a smartphone and or access to internet data. However, response rates to weekly email surveys had very high response rates. Thus, the possibility of offering multiple methods of collecting data using a mobile app or email surveys or phone, that caters to the needs of each participant, may significantly improve response rates and the feasibility of collecting weekly outcomes [58]. Frequent weekly data collection with a large number of participants would be very expensive and bothersome without the use of technology. Another limitation of this study was the fact that it was a one-arm study. This methodology only allows us to evaluate the feasibility of the intervention but not the methods of an RCT where patients that are randomized to the usual care group might have greater issues with retention and adherence with our data collection.

## Conclusion

The results of this study demonstrate an intervention that is feasible and a data collection protocol that can be easily improved to reduce attrition rates. Primary strengths of the pilot study were use of a design that was registered a priori and recruitment of an adequate number of participants to provided relevant feasibility information about the new *Back to Living Well* program and suggestions for improvement. Limitations included the poor tracking of attendance during exercise sessions and suboptimal response rates to the weekly pain measures which provides valuable information for a fully powered study. In general, the program was feasible, with high participant satisfaction. While there was a trend towards improvement of pain, disability, function, quality of life and physical activity over time, the effectiveness of the intervention is unclear given the lack of power. Future high-quality, adequately powered randomized controlled trials are needed to test the effectiveness of this intervention.

## Supplementary Information

**Additional file 1.** End of study satisfaction questionnaire.

**Additional file 2.** CONSORT 2010 checklist of information to include when reporting a pilot or feasibility trial*

## Data Availability

The data are available upon request.

## Abbreviations

LBP: Low back pain
PSFS: Patient-Specific Functional Scale
VAS: Visual analogue scale
EQ-5D-5L: EuroQol
RMDQ: Roland Morris Disability Questionnaire
MCID: Minimal clinical important difference
HHS: Hamilton Health Sciences
YMCA HBB: YMCA Hamilton, Burlington, and Brantford
