# Supplementary figures and images for: Back to living well: community-based management of low back pain: a feasibility study
Source: Pilot Feasibility Stud. 2021 Jun 24;7:134. doi: 10.1186/s40814-021-00863-7 (PMC8223312; doi:10.1186/s40814-021-00863-7)

**Appendix 1**

**
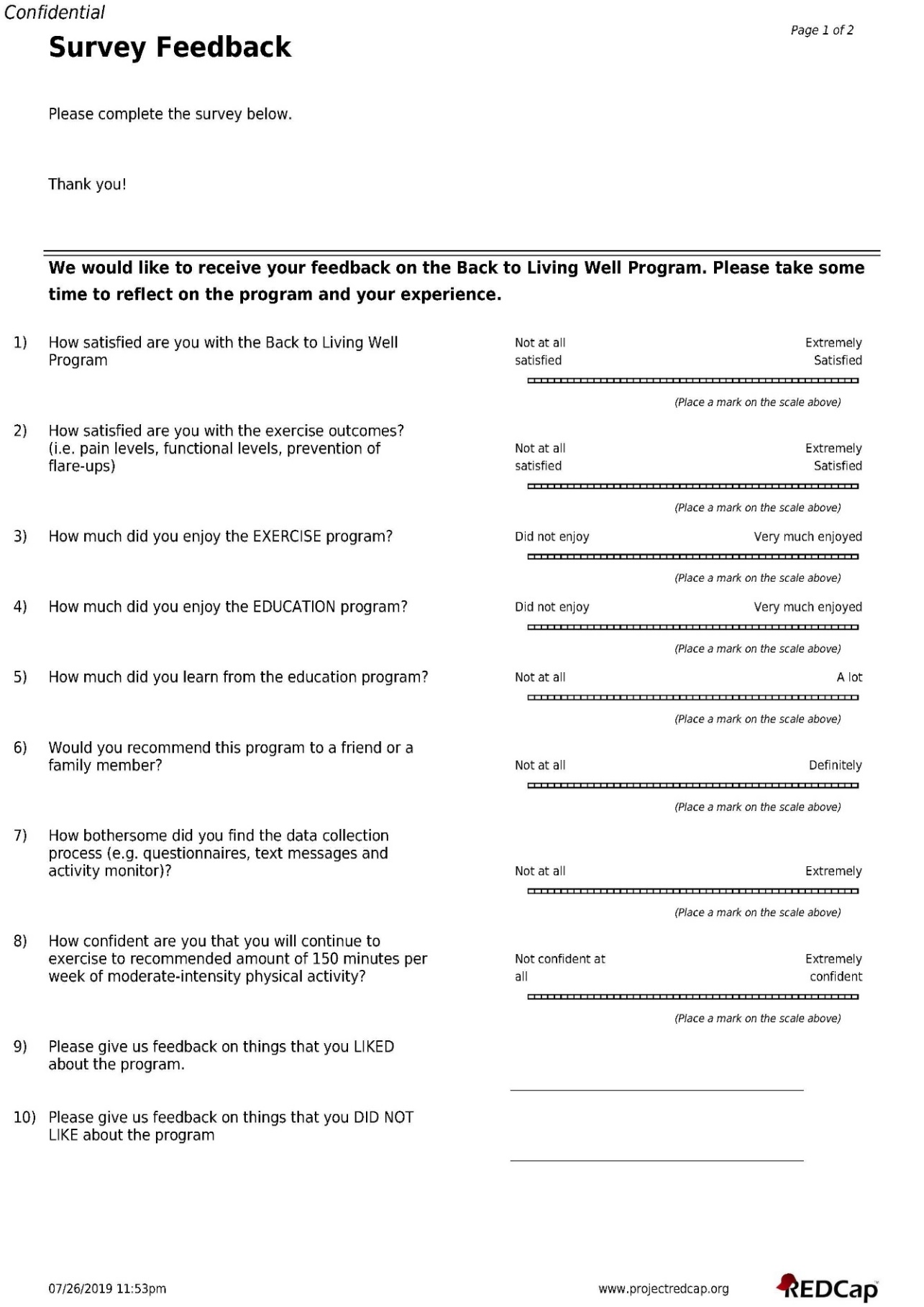
**


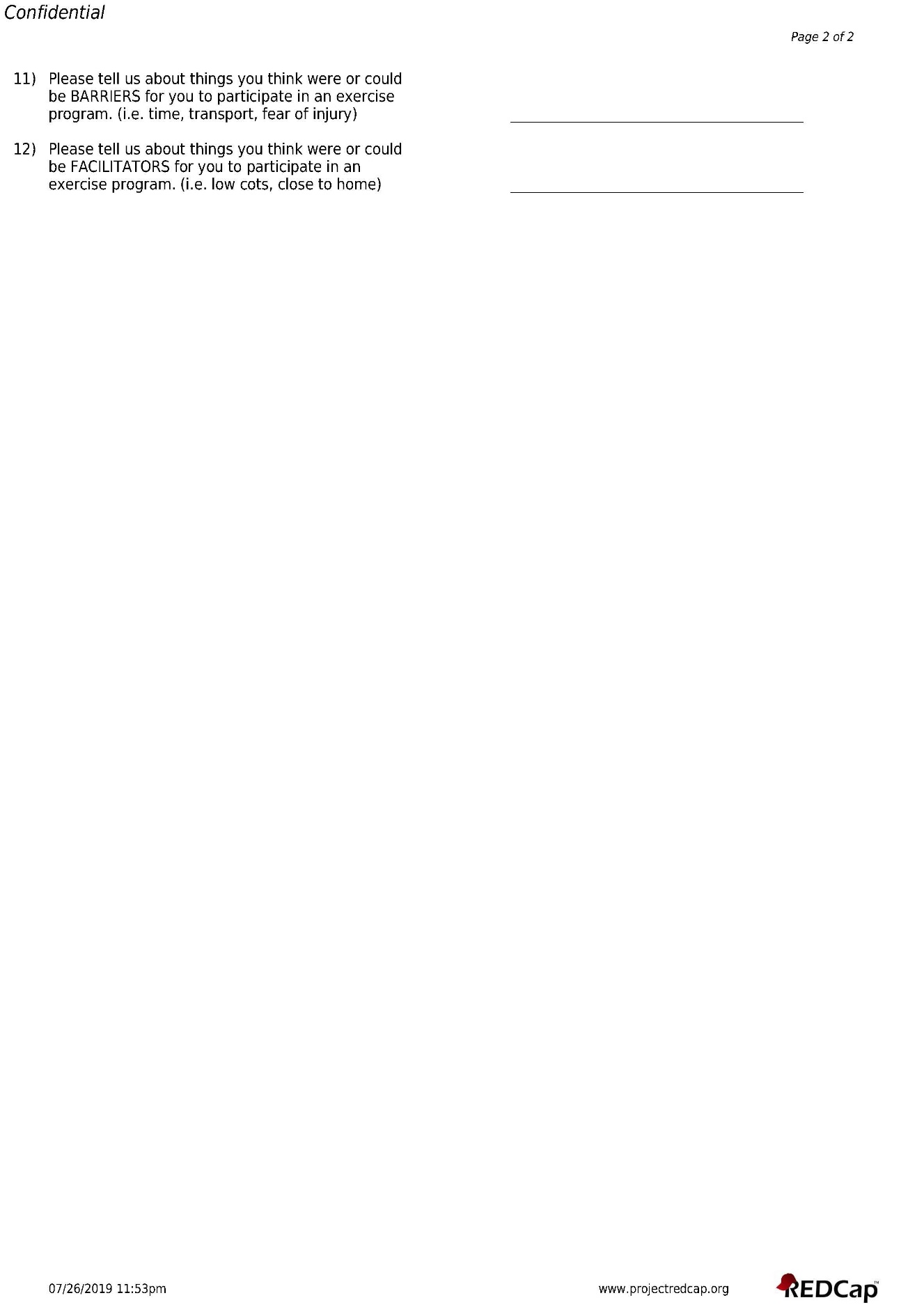

Supplement: Supplementary file 1 — Additional file 1. End of study satisfaction questionnaire. [file 40814_2021_863_MOESM1_ESM.docx]
